# Human preferences toward algorithmic advice in a word association task

**DOI:** 10.1038/s41598-022-18638-2

**Published:** 2022-08-25

**Authors:** Eric Bogert, Nina Lauharatanahirun, Aaron Schecter

**Affiliations:** 1 Department of Supply Chain and Information Management, Northeastern University, Boston, MA 02115 USA; 2 Departments of Biomedical Engineering and Biobehavioral Health, Pennsylvania State University, University Park, PA 16802 USA; 3 Department of Management Information Systems, University of Georgia, Athens, GA 30602 USA

**Keywords:** Human behaviour, Computer science, Information technology

## Abstract

Algorithms provide recommendations to human decision makers across a variety of task domains. For many problems, humans will rely on algorithmic advice to make their choices and at times will even show complacency. In other cases, humans are mistrustful of algorithmic advice, or will hold algorithms to higher standards of performance. Given the increasing use of algorithms to support creative work such as text generation and brainstorming, it is important to understand how humans will respond to algorithms in those scenarios—will they show appreciation or aversion? This study tests the effects of algorithmic advice for a word association task, the remote associates test (RAT). The RAT task is an established instrument for testing critical and creative thinking with respect to multiple word association. We conducted a preregistered online experiment (154 participants, 2772 observations) to investigate whether humans had stronger reactions to algorithmic or crowd advice when completing multiple instances of the RAT. We used an experimental format in which subjects see a question, answer the question, then receive advice and answer the question a second time. Advice was provided in multiple formats, with advice varying in quality and questions varying in difficulty. We found that individuals receiving algorithmic advice changed their responses 13$\%$ more frequently ($\chi ^{2} = 59.06$, $p < 0.001$) and reported greater confidence in their final solutions. However, individuals receiving algorithmic advice also were 13$\%$ less likely to identify the correct solution ($\chi ^{2} = 58.79$, $p < 0.001$). This study highlights both the promises and pitfalls of leveraging algorithms to support creative work.

## Introduction

With rapid advances in artificial intelligence (AI) and computing power, people are increasingly receiving real-time input from algorithms—which we will refer to as algorithmic advice—in place of or in addition to information from other humans. Current algorithms provide recommendations for well-defined problems with readily available data to a broad array of stakeholders. For example, individuals regularly use platforms such as Spotify, Netflix, or Match.com to find music, movies, or romantic partners. Algorithms can also be used to predict the future price of a stock^1,2^, or even anticipate recidivism and set bail^3,4^. A common feature of these scenarios is the objective nature of the outcomes; in every case, the algorithm is providing advice with the intent of helping the decision maker(s) reach an optimal decision. While the algorithms may be incorrect, or even biased, they are tuned to provide a “correct” answer to an underlying optimization problem.

However, this paradigm does not necessarily apply when the task involves creative thinking. It is not clear how algorithmic advice will be received when the objective of a task is to produce and evaluate novel ideas. Here we ask the question, can algorithms help humans to be more creative? More precisely, are humans receptive to algorithmic advice when completing tasks such as brainstorming or word association that require creative thinking? Answering this fundamental question is critical with respect to our society’s increased development and reliance on intelligent technologies. Future businesses, groups, and consumers will be faced with receiving information or advice from intelligent systems or even intelligent teammates^5,6^ to engage in dynamic problem solving with significant ambiguity.

There are often multiple ways to solve complex problems, and a degree of creative thinking is required even when there is an optimal solution. To find a satisfactory solution, people engage in a sequence of idea creation and idea refinement, also referred to as divergent and convergent thinking^7,8^. Divergent thinking is more often associated with creative enterprise as it involves the generation of novel ideas and expansion of the problem space. However, convergent thinking—the evaluation of novel ideas—is also an important component of creative thought. Without convergent thinking, the limitless ideas generated through divergent thinking cannot be assessed for their feasibility or relevance to the problem at hand^8,9^. In this study we focus on how algorithms can aid creativity by supporting convergent thinking.

The extant literature provides mixed answers regarding algorithms and creativity. On one hand, the phenomenon of algorithmic aversion is well documented^10–14^. People tend to reject algorithmic advice in scenarios that are considered more subjective or sensitive, when they have unreasonable expectations of the algorithm’s ability, or when they lack autonomy over their decisions^10^. Given the subjective nature of creative tasks such as brainstorming, humans could be predisposed to reject suggestions from an algorithm. On the other hand, there have been well-documented recent examples of machine learning algorithms identifying novel innovations based on analyses of text data^15^. Further, algorithms are increasingly used to generate text, ranging from simple applications such as auto-complete to more complex programs like GPT-3^16^, BERT^17^, and Transformers^18^. Considering the growing prevalence of “creative” algorithms coupled with their relative efficiency, it is also possible that humans will be biased to accept the advice of algorithms.

Prior literature has demonstrated that receiving advice can significantly boost confidence for a decision maker^19,20^, even to the point of potential overconfidence^21^. Further, decision makers tend to differentiate between good and bad advice, favoring advice sources that are perceived as high quality^22–24^. While it is not clear if advice from an algorithm functions differently, prior work has demonstrated a consistent automation bias among humans using intelligent systems, leading to significant complacency^25–28^. As such, we anticipate a consistent effect, with humans feeling more confident in decisions supported by algorithmic advice. However, it is unclear how humans will react to low-quality advice, given the tendency to punish algorithms for making mistakes^1,10,12,13^.

In this paper we advance our knowledge of this problem. We conducted a preregistered online experiment ($N=154$ participants, 2772 observations) to determine whether humans had stronger reactions to algorithmic advice or social influence when completing multiple instances of the remote associates test (RAT)^29^. The RAT task is an established instrument for determining convergent and creative thinking with respect to multiple word association^30–32^. We specifically tested three research questions. First, to what extent will people incorporate algorithmic advice—compared to advice from other humans—when solving remote association test problems? Second, will advice from algorithms or other humans lead to greater accuracy when solving RAT problems? Finally, do people who receive algorithmic advice express more confidence in their decisions, compared to those who receive advice from other people?

## Results

**Figure 1:**
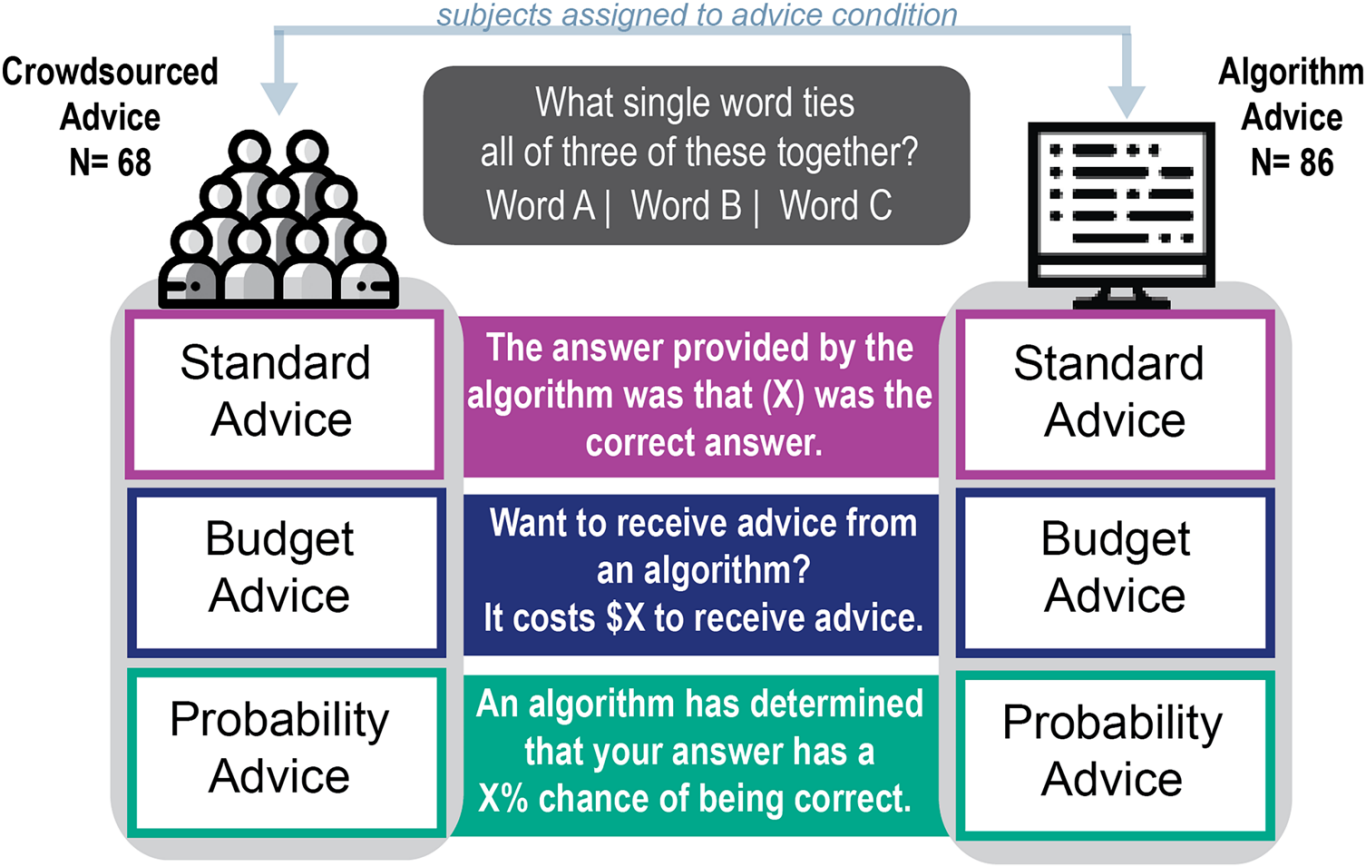
Schematic of experimental procedure.

All subjects answered 18 Remote Associates Test (RAT) questions. We used the Judge Advisor System task format^33^, in which subjects see a question, answer the question, then receive advice and answer the question a second time. Participants are randomly assigned to one of two conditions: algorithmic advice or crowd advice. Advice was given in one of three formats in random order. We varied the advice format to test whether certain forms (a direct suggestion, a probability, or an option to purchase advice) had different effects on behavior and confidence. Participants answer all six questions within one advice condition before moving to the next type. Advice is either of high or low quality (randomized), with half of the questions within each type being high or low quality. RAT questions are either medium, hard, or very hard, and the difficulty is distributed across all conditions in random order. We measure the rate at which participants change their answers after receiving advice, the rate at which they identify the correct response, and the change in their solution confidence. We summarize our experimental format in Fig. 1.

Our primary treatment concerned the source of advice, as perceived by participants. There were two conditions. In one condition, participants were told that they were receiving advice from an algorithm trained on similar problems (treatment group). In a second condition, participants were told that they were receiving the consensus guess of other humans (control group). We verified the salience of the condition using manipulation checks. The source of advice was a between-subjects treatment, while all other features of the experiment such as difficulty, advice format, and advice quality were within-subjects factors. See the methods section for additional information on the procedure.

### People were more likely to incorporate algorithm advice into their decisions

**Figure 2:**
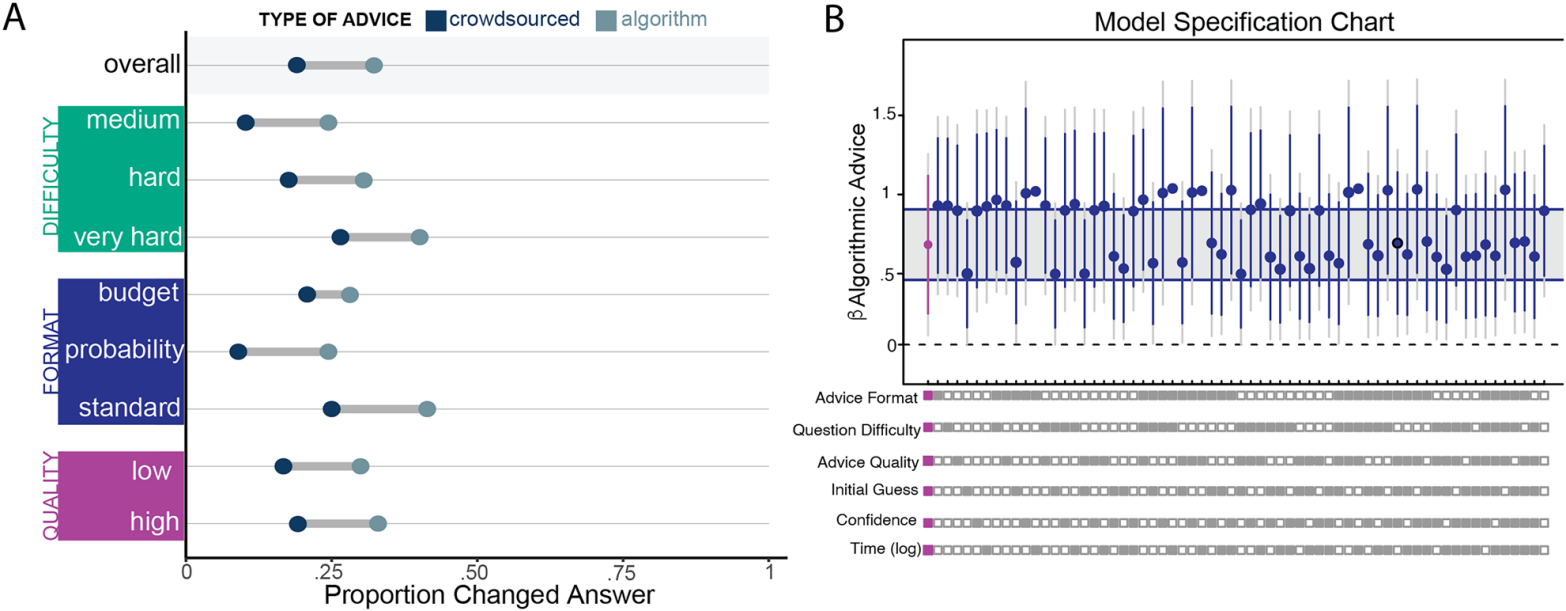
Effect of advice source on acceptance of advice. (**A**) Percentage of observations where individuals changed their answer after being provided with advice. The overall rate encompasses all 2772 observations. Each subsequent comparison filters the data by advice format, question difficulty, or advice quality. All differences are statistically significant using Tukey multiple comparison tests at the 95% confidence level. (**B**) Specification chart illustrating estimates of the coefficient for the effect of algorithmic advice on whether participants changed answers. Each model has a varying number of control variables. The boxes below the chart indicate which controls are included (filled box) or excluded (empty box). The main result is highlighted in purple and contains all controls. All estimates are produced by mixed effects logistic regression models with random slopes for the participants. Intervals are 95% and 99% confidence intervals for the estimates; the grey band is one standard deviation above and below the main estimate.

Our first objective was to differentiate the effect of algorithmic advice from social advice across various conditions. We determined that participants relied upon advice if they changed their answer from their initial guess. Overall, we find that algorithmic advice prompted changes in initial responses more frequently than social advice, with $p_{A} = 0.31$ for algorithmic advice and $p_{S} = 0.18$ for social advice across 2772 responses. This difference is statistically significant using a one-sided test for differences in proportions ($\chi ^{2} = 59.06$, $p < 0.001$). Further, we conducted a Tukey multiple comparison test to account for the various factors in our data (Advice Type, Difficulty, Advice Quality) which could affect the significance of our results. Using a family-wise 95% confidence level, we confirmed that there is a positive and significant effect of algorithmic advice on the likelihood of changing an answer. The Tukey adjusted confidence interval for the difference in rates is (0.097, 0.160). The rates of taking algorithmic advice across all conditions are presented in Fig. 2A.

We next dug deeper into the data and calculated these rates while accounting for advice formats, question difficulty, advice quality, whether or not participants correctly responded on their first guess, their level of confidence in their response, and the time it took them to produce an answer. We applied multilevel modeling to account for the nested nature of the data (multiple rounds within individuals). Because the response variable is binary (Changed: 1, Not Changed: 0), we used a mixed-effects logistic regression model with errors clustered on the individuals. The treatment variable is a binary variable indicating whether participants were in the algorithmic advice condition or not.

We found that the coefficient for the treatment variable was positive and significant in the main model with all controls ($b = 0.654$, $SE=0.231$, $p < 0.01$), indicating a tendency for individuals in the algorithmic advice group to change their responses more often after receiving advice. In context, a coefficient value of 0.654 corresponds to an odds ratio of 1.923, indicating that participants in the algorithmic advice group were 92.3% more likely to change their responses than those in the social advice condition, after accounting for between-subject differences and other controls.

To confirm the robustness of our results to different subsets of control variables, we ran a total of 63 additional mixed-effects logistic regression models. Each model included a different combination of control variables. In Fig. 2B we present the coefficient estimates and confidence intervals for the main treatment variable, algorithmic advice. The results suggest that algorithmic advice prompts people to change their answers significantly more frequently than social advice, regardless of advice format, question difficulty, advice quality, or an individual’s initial response.

### People using algorithm advice were less likely to find the correct answer

**Figure 3:**
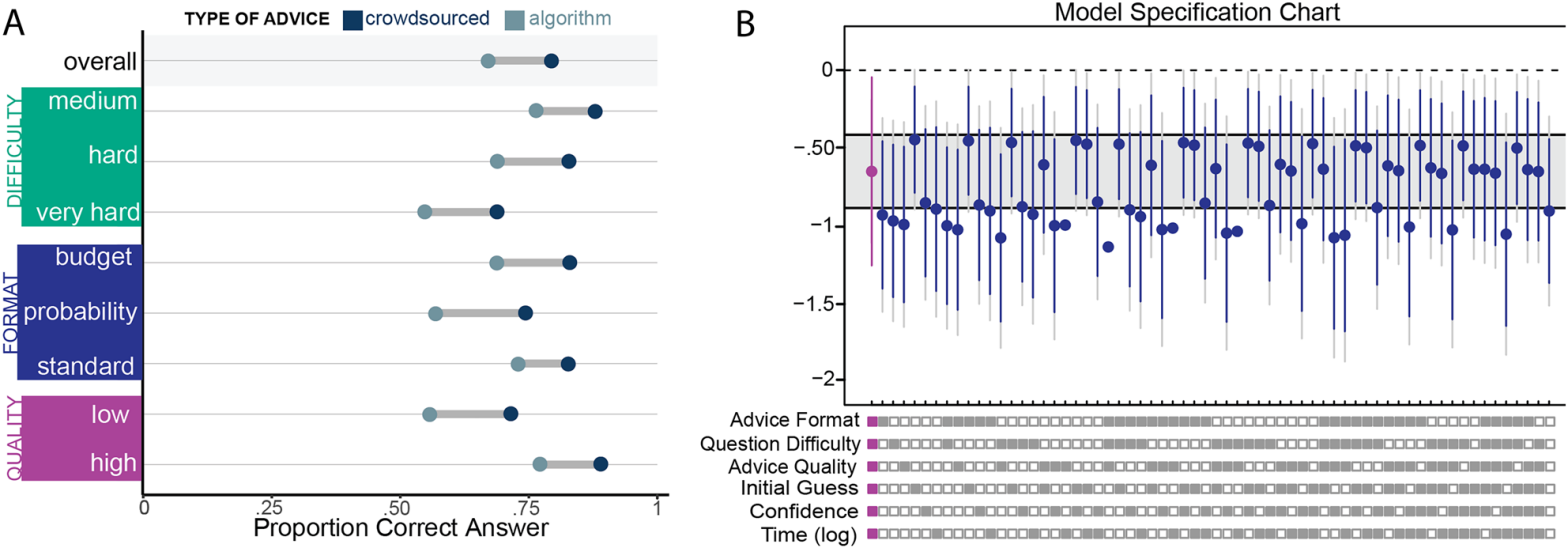
Effect of advice source on rate of identifying correct answer. (**A**) Percentage of observations where individuals identified the correct answer after being provided with advice. The overall rate encompasses all 2772 observations. Each subsequent comparison filters the data by advice format, question difficulty, or advice quality. All differences are statistically significant using Tukey multiple comparison tests at the 95% confidence level. (**B**) Specification chart illustrating estimates of the coefficient for the effect of algorithmic advice on whether participants identified the correct answer. Each model has a varying number of control variables. The boxes below the chart indicate which controls are included (filled box) or excluded (empty box). The main result is highlighted in purple and contains all controls. All estimates are produced by mixed effects logistic regression models with random slopes for the participants. Intervals are 95% and 99% confidence intervals for the estimates; the grey band is one standard deviation above and below the main estimate.

We next determined whether or not exposure to algorithmic advice helped people correctly identify the solution to the RAT problems. Overall, participants identified the correct answer 65% of the time on their first guess, and 72.3% of the time after receiving advice. This 7.2% improvement in accuracy is statistically significant using a test for equality of proportions ($\chi ^{2} = 34.55$, $p < 0.001$), indicating that advice does indeed help participants find the correct answer. Further breaking this rate down by advice source, we find that individuals who received algorithmic advice only produced a final correct answer 66.54% of the time, compared to 79.66% of the time for those receiving social advice. This difference is also statistically significant ($\chi ^{2} = 58.79$, $p < 0.001$), implying that people using algorithmic advice were less likely to correctly identify the RAT solution. We also conducted a Tukey multiple comparison test to account for the impact of other factors on the significance of our findings. Using a family-wise 95% confidence level, we confirmed that there is a negative and significant effect of algorithmic advice on the likelihood of finding a correct answer. The Tukey adjusted confidence interval for the difference in correct answers is ($-0.163, -0.100$), indicating that people using algorithmic advice were right significantly less often. The differences in rates of identifying the correct answer are presented in Fig. 3A; all differences are statistically significant at the 95% confidence level.

To more rigorously test the effect of advice source on accuracy, we again used multilevel logistic regression (Final Answer Correct: 1, Incorrect: 0) with errors clustered on the individual participants, and the advice source condition the binary treatment variable. We found that individuals who received advice from an algorithm, rather than other people, were significantly less likely to submit a final correct solution ($b = -0.654$, $SE=0.236$, $p < 0.01$). This coefficient suggests that people receiving algorithmic advice were 48% less likely to produce the right answer than people who received advice from other people. This result is robust across various subsets of control variables, as we show in Fig. 3B.

However, these analyses do not account for the fact that some individuals never changed their answer, and that people changed their answers at different rates depending on the type of advice they receive. Both of these issues potentially threaten the validity of our analyses. To remedy this problem, we focus only on instances where the participant changed their answer $(N=708)$. Within this sample, participants receiving algorithmic advice identified the correct answer 171 times out of 483 observations (35.4%) compared to 109 times out of 226 observations (48.4%) for social advice. This difference in proportions is statistically significant ($\chi ^{2} = 10.92$, $p < 0.001$). Finally, we considered the possibility that people are getting the wrong answer because they are changing their guess after initially finding the correct solution. Because we want to determine if algorithmic advice can specifically help people find a correct solution, we further reduced our dataset to only cases where the participant (a) made an incorrect initial guess and (b) changed their answer after receiving advice $(N=631)$. Among this subsample, we find that people receiving algorithmic advice were correct 171 out of 431 times (39.7%), while people receiving social advice were correct 109 out of 200 times (54.5%). Again, this difference is statistically significant ($\chi ^{2} = 12.16$, $p < 0.001$), indicating that people using algorithmic advice are less likely to find the correct answer to a RAT question.

### People indicated higher levels of confidence after receiving advice from algorithms

Participants who received advice from algorithms—rather than other people—also reported higher levels of confidence in their final solutions, regardless of their initial confidence.

**Figure 4:**
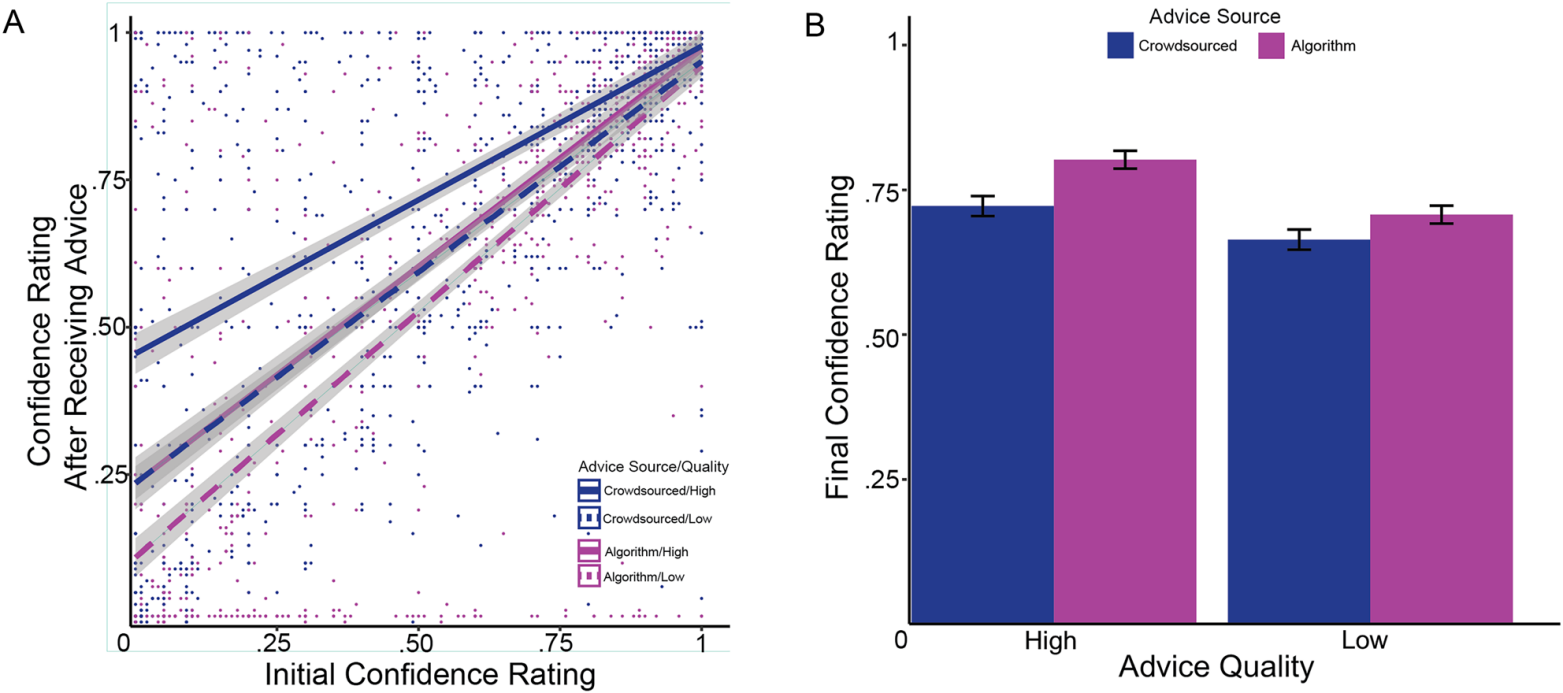
Relationship between advice source, quality of advice, and confidence. (**A**) The relationship between participants’ Initial Confidence and Final Confidence, broken down by advice source and quality. Purple lines indicate algorithmic advice, and solid lines indicate high quality advice. All lines are linear best fits with 95% confidence bands. (**B**) The estimated marginal mean Final Confidence, accounting for initial confidence. Error bars represent one standard deviation. All pairwise differences between marginal means are statistically significant at $p<0.01$ using Tukey’s multiple comparison criterion.

In Fig. 4A we demonstrate how initial confidence and post-response confidence are related, accounting for advice condition and the quality of advice. In general, people become more confident after receiving advice; this fact follows from the trend lines being above a hypothetical 45 degree line. When the quality of the advice is held equal, receiving advice from an algorithm tends to produce a larger increase in confidence compared to social advice, as evidenced by the purple lines lying above the blue lines. Further, people also tend to gain more confidence when the quality of advice is high compared to low, reflected in the solid lines lying above their corresponding dashed lines.

To test whether the advice source and quality of advice had significant effects on final confidence, we conducted an analysis of covariance (ANCOVA). ANCOVA is an appropriate method given the presence of a continuous covariate (Initial Confidence) that has a linear relationship with the outcome variable. We find that advice source $(F=45.50, p<0.001)$, quality of advice $(F=21.49, p<0.001)$, and their interaction $(F=4.80, p<0.05)$ all explain significant variance in Final Confidence after taking Initial Confidence into account. We next calculate the estimated marginal means and their standard errors from the ANCOVA model. Finally, we test for differences across the estimated means using a Tukey multiple comparison test, with a family-wise 95% confidence level. The results for both the mean estimates and absolute differences are presented in Fig. 4B. We find that all differences are statistically significant with an adjusted *p* value of less than 0.01 in all cases except for (Crowd, High Quality) and (An Algorithm, Low Quality), which were not significantly different.

## Discussion

In this study, we find evidence that advice coming from an algorithmic source—rather than other people—significantly influences how individuals solve analytic problems. Our approach differs from many modern use cases of algorithmic input—such as movie recommendations on Netflix—in that we explicitly inform participants of the advice source. By doing so, we attempt to highlight how humans behave differently if *they believe* they are receiving input from an algorithm. We specifically investigate the role of algorithmic advice on a word association task that measures certain elements of creativity, the remote associates test. The RAT task is an established method for assessing both creative and analytic thinking^29–32^, and to our knowledge this is the first study to test the effect of algorithmic advice on this type of task. Our experiment reveals that humans behave in two key ways, even after controlling for how advice is given, how difficult the task is, and whether or not the advisor provides accurate advice. First, individuals who received algorithmic advice revised their answers significantly more often than those who received social advice. Second, individuals who received algorithmic advice were significantly less likely to correctly solve a RAT question. This pattern holds even when isolating individuals who started with an incorrect response and changed their answer when receiving advice. In other words, when the advice provided was beneficial, people still performed better when receiving the information from a purported human source. In sum, humans are more likely to change their responses when receiving advice from an algorithm, even though on average they are less successful.

We also examined the effect of advice source on the change in reported confidence. Individuals receiving algorithmic advice reported significantly higher confidence after their second guess than individuals in the social condition. They also reported greater confidence when the advice provided was of high quality—i.e., a verifiable correct answer. This difference is particularly salient when the algorithm provides high quality advice; here participants reported the highest confidence levels. However, when an algorithm provides bad advice—an incorrect answer—individuals in the study reported confidence levels on par with correct human advice. This finding suggests that while humans do penalize an algorithm for erring, as shown in prior work^12^, they also feel generally more confident when their advice is algorithmic, regardless of its quality. A potential rationale for this finding is that humans are willing to overlook mistakes by AI if they are able to modify the solutions^13^—in this case, use their own answers to the RAT task.

These findings reveal a conundrum in designing cooperative human-machine hybrid systems^6,34,35^. On one hand, humans are responsive to algorithmic advice. On the other hand, algorithmic advice may not improve human performance. Coupled with the boost in confidence experienced by individuals who received algorithmic advice, we posit that our task elicited a form of automation bias^25–28^. Namely, humans rely upon a machine—or a purported algorithm in our case—to perform repeated tasks and over time become complacent. As a result, they may not catch cases where the machine errs, or place undue faith in the machine’s capabilities. Given the increasing prevalence of human-machine collaboration, we argue that designers of these hybrid systems should be cognizant of the potential for this type of automation bias. Further, the results suggest that simply placing a “human-in-the-loop” may not be sufficient to counter errors produced by algorithms, particularly when the system has a history of high performance.

The results also provide further evidence for algorithmic appreciation, rather than aversion, in tasks with measurable outcomes. Individuals have shown a tendency to favor algorithms when solving problems involving logic^36^, or when making quantitative judgements under uncertainty^37,38^. Conversely, aversion is typically strongest in subjective or open-ended tasks^10,11,14,39^. Even in quantitative tasks, appreciation is not guaranteed^12,13^. In fact, the notion of a “spectrum” of objectivity has been noted in a review of human willingness to accept algorithmic advice^11^. Consequently, we anticipated that a task such as the RAT would arouse some degree of aversion. That we found a consistent appreciation effect suggests to us perhaps word association tasks, while more subjective than tasks such as counting or forecasting, are still relatively analytical in how humans solve them^40,41^. Indeed, there is some debate regarding the relationship between the RAT task and creativity. Recent scholarship has suggested that the RAT is a better measure of *convergent* thinking, i.e., confirming the validity of an answer, rather than divergent thinking which involves generating new possibilities^40,41^. However, the RAT task still requires individuals to make free associations between words, and people who are better at the RAT task are less likely to fixate on common words^42^. Thus, while the RAT test is likely a more analytical task than other creative tasks, it still captures an important part of the creative process^40^. It is also relevant to some ways in which algorithms are used in practice, such as smart assistants and other text-based automation tools deployed to improve worker productivity. Thus, while our results may not predict algorithmic appreciation (or aversion) in all creative tasks, they do suggest that humans are willing to accept text-based advice from algorithms in certain circumstances, albeit with the risk of automation bias and complacency. Future work is needed to explore how humans react to algorithms for tasks requiring more significant divergent thinking, such as the alternative uses task^43^.

Finally, we consider the implications for human-autonomy trust. Prior work has explored the antecedents of trust in autonomous agents^44–46^ and the role of trust in fostering effective interactions. One particular dimension of trust—performance-based—refers to the confidence that a human develops towards an autonomous agent on account of its demonstrated ability to perform a task successfully. This is akin to what is referred to in psychology as cognitive trust^47^. Various aspects about the machine’s performance, such as its predictability^48,49^, transparency^45,50^, and, reliability^50–52^ affect the extent to which humans will trust that machine. Other work has suggested that trust is task-dependent; when solving problems involving logic or quantitative judgements, participants trusted the same solution more when it came from an expert system (algorithm) relative to receiving the solution from a human^36,38^. In our experiment, we found that humans seemed inclined to trust the autonomous agent, i.e., they relied on the algorithm for advice, despite having no information regarding its predictability or performance. This finding has important implications for future human-autonomy interactions. For example, when humans are faced with logic-based tasks (not necessarily quantitative), we anticipate that they will be more likely to trust in decision support received from an algorithmic rather than a human advisor. This potential reliance may be problematic since it is disconnected from performance appraisals. In our experiments, people leveraged algorithmic advice even when that advice was incorrect. Depending on the decision-making context, this preference for relying on algorithmic advice in the face of uncertainty may not be in service of optimal performance. Accordingly, individuals or organizations designing human-autonomy hybrid systems should be aware of these inherent biases for a broad range of tasks, including certain types of creative tasks.

## Methods

This study was approved by the University of Georgia Institutional Review Board, project 00001012. Subjects gave written informed consent both before and after participation in the study. All methods were carried out in accordance with relevant guidelines and regulations.

### Participant recruitment

Participants were recruited from Amazon Mechanical Turk (AMT). All subjects had a greater than 98% lifetime approval rate for prior tasks conducted on AMT, had completed at least 500 tasks on AMT, were located in the United States, and could only participate in the task once. We started with 182 respondents recruited from AMT. Of those, 14 failed the manipulation check, and an additional 14 provided incomplete responses or did not enter a valid MTurk identification value. The analysis is based on the 154 remaining subjects, compared with the preregistered plan of at least 148 subjects. Each subject was paid USD 5.00 to complete the experiment, and an additional bonus of USD 1.00 was given to subjects who correctly answered a randomly selected question in each of the conditions, allowing for up to USD 3.00 of bonuses per subject based on their performance in the RAT questions. Subjects were told the study would take approximately half an hour to complete.

### Task and main manipulation

All subjects saw 18 RAT questions. In a RAT question, there are three words that are tied together by a fourth word, which can come before or after each of the three words (see Supplementary Information for the list of RAT questions and solutions). For example, participants were shown *Base*, *Room*, and *Bowling*, the answer would be **BALL** (Base**Ball**, **Ball**Room, Bowling **Ball**).

The fourth word can come before or after each of the three words, and can either form a compound word (e.g., ballroom) or a common two-word expression (e.g., bowling ball). We selected remote associates test questions from a well-known repository of remote associates test questions that also includes how difficult each question is^53^. For each question, the correct answer was the answer offered by the remote associates test. The wrong answer was created by the authors. Each wrong answer fits two of the three given words. Difficulty was predetermined based on the average time it took individuals to solve the RAT question historically. The questions used in this study ranged in difficulty from medium to very hard. For a list of the questions and answers, as well as details on generating wrong answers, please see the Supplementary Information.

After each question, participants were asked to rate their confidence in their answer. Then, participants were shown some form of advice, after which they were able to change their answer. They then rate their confidence a second time.

Subjects were randomly assigned to the social or algorithmic condition, which was a between-subjects condition throughout the experiment—subjects only received advice from one source throughout all 18 questions. In the instructions, participants in the social condition saw an advice source that read: “the consensus of other people who have completed this task.” Participants in the algorithmic condition saw an advice source that read: “the output of an algorithm that has been trained to solve similar word association tasks.” The specific delivery format of the advice was varied across the questions. As a manipulation check, at the end of the survey, we asked subjects “What was the source of advice you received in this survey?” with three answer choices: “other people”, “an algorithm”, and “I don’t remember.” Fourteen subjects chose either the “I don’t remember” option or the option that did not correspond to the type of advice they received. We excluded those subjects from our analysis.

### Advice format conditions

After making an initial guess, participants were then provided with advice, or the opportunity to receive advice. Each subject saw three types of questions: Probability, Standard RAT, and Budget. Subjects saw all questions of one type, then all questions of another type, with no mixing of the types. For example, a subject would see all six Probability questions, then all six Standard RAT questions, then all six Budget questions. Within each advice type, two questions were medium, two were hard, and two were very hard. Before each group of questions, we would provide additional instructions on how the advice would be delivered.

For the standard RAT group of questions, subjects always received advice after answering a question. This case follows the typical judge advisor system format. For the Probability group of questions, subjects were asked a RAT question and gave an answer. Then, subjects were shown a probability that their answer was correct; this value was influenced by their first response. For the Budget group of questions, we offered subjects the chance to buy advice for ten cents. This value was significantly lower than the potential bonuses for correct answers, and was thus meant to encourage people to try and increase their likelihood of a higher payout. If they purchased advice, it was then provided in the same way as the standard RAT case. In all cases, the prompt was modified by the algorithmic condition.

### Advice quality

Within a block of six questions, each subject saw high-quality advice three times and low-quality advice three times. High- and low-quality advice was evenly distributed across advice type and question difficulty, in random order. For high quality advice, participants were shown the correct answer. For low quality advice, the clue fit two of the three words, but not all three. For example, our wrong answer advice for a remote associates test question for the words “Cross”, “Rain” and “Tie” was “Hair” because it fit “Cross” (Cross hair) and “Tie” (Hair tie) but not “Rain.” The correct answer is “Bow.” We used a third-party dictionary-based website to validate that our wrong answers fit exactly two of the three words.

## Supplementary Information

Supplementary Information.

## Data Availability

The data sets analysed during the current study, as well as code to conduct analyses, are available from the corresponding author on request.
