# Supplementary material for: Human preferences toward algorithmic advice in a word association task
Source: Sci Rep. 2022 Aug 25;12:14501. doi: 10.1038/s41598-022-18638-2 (PMC9411628; doi:10.1038/s41598-022-18638-2)
Supplement: Supplementary file 1 — Supplementary Information. [file 41598_2022_18638_MOESM1_ESM.docx]

**Supplementary Information for**

Human Preferences Toward Algorithmic Advice in a Word Association Task

Eric Bogert

Nina Lauharatanahirun

Aaron Schecter

Corresponding Author: Aaron Schecter

Email: [aschecter@uga.edu](mailto:aschecter@uga.edu)

Experimental Details

We preregistered our experiment with the Open Science Foundation [here](https://osf.io/fwb4x/?view_only=f692304d882c4416aa181a3fe80726d1).

**Participant Interface and Sample Questions.** Each subject saw a question that said “What single word ties all three of these together?” followed by the three words for the question. We recorded how long subjects spent answering this question. Then, subjects were sent to a new page and asked how confident they were in their answer. Depending on the question group subjects were in during the survey, subjects then saw a follow-up question with advice. For the questions in the Standard advice group, subjects were simply brought to a new page and displayed the text seen below in Table S1. For questions in the Probability group, subjects saw the text in the Probability row below. An explanation of the X variable for the probability questions follows the table. For questions in the Budget group subjects saw a screen after the initial question asking about confidence. Subjects in the algorithmic advice group saw “Do you want to receive advice on this question? It costs ten cents to receive advice.” With two options: “I want to receive advice from an algorithm” and “I don’t want to receive advice.” Subjects in the social advice group saw the same question and answer, but instead of seeing “I want to receive advice from an algorithm” they saw “I want to see advice from other people.” If subjects opted out of the advice, they were simply asked the question again.

**Manipulation and Attention Checks.**

At the end of the survey, we asked subjects “What was the source of advice you received in this survey?” with three answer choices: “other people”, “an algorithm”, and “I don’t remember.” Fourteen subjects chose either the “I don’t remember” option or the option that did not correspond to the type of advice they received. We excluded those subjects from our analysis.

**Design of Probability Questions.** When subjects answered one of the six questions in the Probability group, they saw a percentage related to how good their answer was. Subjects in the algorithmic condition saw text that read "An algorithm has determined that your answer has an {X}% chance of being correct." Subjects in the social condition saw text that read "{X}% of other people who took the survey gave an answer similar to yours." If the question gave low quality advice, meaning that it gave the wrong answer as advice, then the subject also saw a range of percentages that were different and non-overlapping with the range of percentages when subjects saw high quality, correct advice. When receiving low-quality advice, if the subject put the correct answer as their answer to the first question, then they saw an X generated on a uniform distribution (see text above) between 51 and 69. If they answered incorrectly, they saw an X generated on a uniform distribution between 31 and 50. For the high-quality advice answers, these percentages were between 70 and 99 for subjects whose first guess was correct, and between 0 and 30 for subjects whose first guess was not correct.

**Details on RAT Questions.** We selected remote associates test questions from a well-known repository of remote associates test questions that also includes how difficult each question is (1). Difficulty was predetermined based on the average time it took individuals to solve the RAT question historically. For each question, the correct answer was the answer offered by the remote associates test. The wrong answer was created by the authors. Each wrong answer fits two of the three given words. We used an external website that captures when English words are used together in phrases to validate that each wrong answer fits exactly two of the words (2). The list of questions, answers, wrong answers, and difficulty are shown in Table S2.

Analyses

**Randomization Checks and Summary Statistics.** In addition to the experimental treatments, we also collected some basic demographic information from participants (Table S3) as well as survey items. Questions included participants’ preferred pronouns and their experience working on Amazon MTurk including tenure, hours per week, and HITS per week. We asked participants to complete the negative attitudes towards robots scale (NARS) to assess possible bias against algorithmic advice. All ten items were included (3). Finally, we asked participants to complete a series of questions regarding the negative impact of the Covid-19 pandemic on their lives. All relevant survey items are included in Table S4. A total of 123 individuals provided this information (78.8%).

In Table S5 we provide the responses to the previously described survey items. For pronouns and MTurk tenure, counts are provided; for all other information the mean and standard deviation is included. To ensure that participants were evenly distributed over the conditions, we conducted two types of tests. For the count variables, we used Pearson’s chi-square test of independence. For the continuous variables we conducted a one-way ANOVA, with advice condition as the independent variable. In all cases we found no evidence of an unequal distribution by condition (p-values greater than 0.10 for all difference statistics). We thus conclude that random assignment was successful.

**Details on Main Models.** For our main analyses, we conducted mixed effects logistic regressions to determine what factors determined whether (i) individuals changed their answers and (ii) whether individuals identified the correct answer. We estimated the following models:

$$a_{it}=\beta_{0}+\beta_{1}z_{i}+\sum_{p} \eta_{p}x_{itp}+\varepsilon_{i}$$

$$c_{it}=\theta_{0}+\theta_{1}z_{i}+\sum_{p} \gamma_{p}x_{itp}+\delta_{i}$$

In the above equations, $a_{it}=1$ if individual $i$ takes advice on question $t$ and 0 otherwise; $c_{it}=1$ if individual $i$ is correct on question $t$ and 0 otherwise; the treatment variable $z_{i}$ is a binary variable indicating whether participant $i$ was in the algorithmic advice condition or not; $x_{ipt}$ is a control variable indexed by participant and question; $\varepsilon_{i}$ and $\delta_{i}$ are random error terms; and $\beta$, $\eta$, $\theta$, and $\gamma$ are coefficients to be estimated. The control variables include design factors – advice type, question difficulty, and advice quality – and question-level behaviors – initial time taken, initial confidence, initially correct.

Table S6 shows the complete results for the analysis of changing answers, and Table S7 shows the complete results for the analysis of identifying the correct answer. We find that the treatment effect coefficients ($\beta_{1}$ and $\theta_{1}$) are consistently positive and negative, respectively, indicating that individuals take algorithmic advice more frequently, but are less likely to find a correct answer. The main models (i.e., Model 3) in both tables correspond to the highlighted models in Figures 2 and 3 in the primary text.

**Robust ANCOVA.** In the main text we analyzed the effect of the advice source treatment on participants’ confidence after providing a second response to each question. Because we are measuring the change in confidence relative to an initial confidence rating, we use an ANCOVA model (see Figure 4 in the main text for results). However, to use an ANCOVA model there are certain assumptions that must be met, including homogeneity of variances and homogeneity of slopes. To verify that our results hold if we relax those assumptions, we conduct a robust ANCOVA (4, 5) using the WRS2 package in R. The output provides estimated differences in the outcome variable – Final Confidence – between levels of the treatment variable – Advice Source – at different values of the control variable Initial Confidence. The results are presented in Table S8 and are broken down by Advice Quality. We see that participants in the Algorithmic advice condition consistently report higher final confidence using robust ANCOVA. At high values of initial confidence, the difference is small and no longer statistically significant, in line with visual inspection of the data. This evidence suggests that our main findings are robust.

**Table S1** Prompts seen by participants in each condition of the experiment.

|  | Social | Algorithmic |
| --- | --- | --- |
| Standard | What single word ties all three of these together? The most common answer for this question provided by **other people** was that {X} is the correct answer.  Word A \| Word B \| Word C | What single word ties all three of these together? The answer provided by **an algorithm** was that {X} is the correct answer.  Word A \| Word B \| Word C |
| Probability | {x}% of **other people** who took the survey gave an answer similar to yours. Based ono this information, you can submit either your old answer or submit a new answer.  What single word ties all three of these together?  Word A \| Word B \| Word C | **An algorithm** has determined that your answer has a {x}% chance of being correct. Please revise your answer, based on this information. You may submit a new answer or keep your old answer.  What single word ties all three of these together?  Word A \| Word B \| Word C |
| Budget | **Other people** have determined that the most likely solution for the three words below is: {X}  Word A \| Word B \| Word C  Given the recommendation of the other people, please write your answer below. | **An algorithm** has determined that the most likely solution for the three words below is: {X}  Word A \| Word B \| Word C  Given the recommendation of the algorithm, please write your answer below. |

**Table S2** RAT questions

| **Clue** | **Correct Answer** | **Wrong Answer** | **Difficulty** |
| --- | --- | --- | --- |
| Fence, Card, Master | Post | Key | Very Hard |
| Mail, Board, Lung | Black |  | Very Hard |
| Sense, Courtesy, Place | Common |  | Medium |
| Keg, Puff, Room | Powder | Ball | Medium |
| Dust, Cereal, Fish | Bowl | Gold | Hard |
| Cat, Right, Carbon | Copy |  | Hard |
| Trip, House, Goal | Field |  | Very Hard |
| Bump, Egg, Step | Goose | Up | Very Hard |
| Pie, Luck, Belly | Pot | Pork | Medium |
| Fox, Man, Peep | Hole |  | Medium |
| Stick, Maker, Point | Match | Bread | Hard |
| Cross, Rain, Tie | Bow | Hair | Hard |
| End, Line, Lock | Dead | Grid | Very Hard |
| Control, Place, Rate | Birth |  | Very Hard |
| French, Car, Shoe | Horn | Door | Medium |
| Flower, Friend, Scout | Girl |  | Medium |
| Time, Blown, Nelson | Full | Half | Hard |
| Tail, Water, Flood | Gate | High | Hard |

**Table S3** Demographics (N=154 responses)

| **Demographic Item** | **Response Count** |
| --- | --- |
| **Age** |  |
| 18-24 | 12 |
| 25-34 | 60 |
| 35-44 | 43 |
| 45-54 | 19 |
| 55-64 | 14 |
| 65-74 | 6 |
|  |  |
| **Education level** |  |
| Associate Degree | 10 |
| Bachelor’s Degree | 69 |
| High School / GED | 18 |
| Master’s Degree | 22 |
| Ph.D. | 2 |
| Professional Degree | 3 |
| Some College | 26 |
| Vocational Training | 4 |
|  |  |
| **Income** |  |
| $0 | 3 |
| $1 to $49,999 | 98 |
| $50,000 to $99,999 | 42 |
| $100,000 to $149,999 | 9 |
| $150,000 and greater | 2 |
|  |  |
| **English Proficiency** |  |
| Full Professional Proficiency | 6 |
| Native/Bilingual Proficiency | 146 |
| Professional Proficiency | 2 |

**Table S4** Survey Items for Covid and NARS measures

| **COVID Impact Items** |
| --- |
| Since the COVID-19 pandemic, I have felt more anxious than usual. |
| Since the COVID-19 pandemic, I have been less social than usual. |
| Since the COVID-19 pandemic, I talk to strangers less. |
| Since the COVID-19 pandemic, I have felt more uneasy around people than usual. |
| Since the COVID-19 pandemic, I have been more likely to avoid physical contact with other people. |
| Since the COVID-19 pandemic, I have worked from home more often. |
| Since the COVID-19 pandemic, I have felt more lonely than usual. |
| Since the COVID-19 pandemic, I take less risks than usual. |
| Since the COVID-19 pandemic, I am more worried that I will get sick. |
|  |
| **NARS Items** |
| I would feel uneasy if I was given a job where I had to use an algorithm |
| The word algorithm means nothing to me |
| I would feel nervous using an algorithm in front of other people |
| I would hate the idea that algorithms or artificial intelligences were making judgements about things |
| I would feel paranoid interacting with an algorithm |
| I would feel uneasy if artificial intelligence really had emotion |
| Something bad might happen if algorithms developed into living beings |
| I feel that if I depend on algorithms too much, something bad might happen |
| I am concerned that algorithms would be a bad influence on children |
| I feel that in the future society will be dominated by algorithms |
| *Notes. All COVID items are on a 7-point Likert scale. The NARS Items are on a 5-point Likert scale.* |

**Table S5** Summary Statistics (N=123 responses)

|  | **Experimental Condition** | |  |
| --- | --- | --- | --- |
|  | Social | Algorithm | Difference |
| **Pronoun Choice:** |  |  |  |
| He/Him/His | 39 | 33 | $\chi_{2}^{2}$ = 1.002 |
| She/Her/Hers | 22 | 27 | p = 0.606 |
| They/Them/Theirs | 1 | 1 |  |
|  |  |  |  |
| **MTurk Experience:** |  |  |  |
| 0-6 Months | 6 | 10 | $\chi_{4}^{2}$ = 7.283 |
| 6-12 Months | 12 | 4 | p = 0.122 |
| 1-2 Years | 9 | 11 |  |
| 2+ Years | 35 | 36 |  |
|  |  |  |  |
| **MTurk Activity:** |  |  |  |
| Hours per Week | Mean = 19.90 | Mean = 19.48 | F = 0.025 |
|  | SD = 13.63 | SD = 15.53 | p = 0.875 |
|  |  |  |  |
| Jobs per Week | Mean = 163.48 | Mean = 305.36 | F = 1.450 |
|  | SD = 214.67 | SD = 901.12 | p = 0.231 |
| **Additional Questions:** |  |  |  |
| NARS | Mean = 2.58 | Mean = 2.60 | F = 0.041 |
|  | SD = 0.77 | SD = 0.62 | p = 0.840 |
|  |  |  |  |
| COVID Impact | Mean = 2.78 | Mean = 2.89 | F = 0.243 |
|  | SD = 1.13 | SD = 1.26 | p = 0.623 |

**Table S6** Mixed Effects Logistic Regression Predicting Change in Answer

| Variable | (1) | (2) | (3) |
| --- | --- | --- | --- |
| Constant | -1.897*** | 0.371 | 2.051*** |
|  | (0.173) | (0.392) | (0.483) |
| Source (Algorithm) | 0.876*** | 0.581** | 0.654** |
|  | (0.218) | (0.207) | (0.231) |
| Initial Confidence |  | -2.015*** | -2.177*** |
|  |  | (0.303) | (0.344) |
| Initial Time (log) |  | 0.146 | 0.298** |
|  |  | (0.084) | (0.094) |
| Initial Correct (Yes) |  | -3.034*** | -4.071*** |
|  |  | (0.201) | (0.258) |
| Advice Quality (Low) |  |  | -0.151 |
|  |  |  | (0.140) |
| Difficulty (Hard) |  |  | -0.051 |
|  |  |  | (0.185) |
| Difficulty (Very Hard) |  |  | -0.284 |
|  |  |  | (0.182) |
| Type (Probability) |  |  | -3.020*** |
|  |  |  | (0.223) |
| Type (Budget) |  |  | -1.861*** |
|  |  |  | (0.208) |
| Random Effect SD | 1.229 | 0.946 | 1.089 |
| N | 2772 | 2772 | 2772 |
| logLik | -1397.801 | -886.873 | -755.054 |
| AIC | 2801.602 | 1785.745 | 1532.109 |
| *** p < 0.001; ** p < 0.01; * p < 0.05. |  |  |  |

**Table S7** Mixed Effects Logistic Regression Predicting Correct Final Answer

| Variable | (1) | (2) | (3) |
| --- | --- | --- | --- |
| Constant | 1.836*** | 0.250 | 2.161*** |
|  | (0.191) | (0.366) | (0.499) |
| Source (Algorithm) | -0.910*** | -0.476** | -0.654** |
|  | (0.238) | (0.178) | (0.236) |
| Initial Confidence |  | -0.090 | -0.198 |
|  |  | (0.281) | (0.348) |
| Initial Time (log) |  | -0.219** | -0.233* |
|  |  | (0.081) | (0.102) |
| Initial Correct (Yes) |  | 4.124*** | 5.929*** |
|  |  | (0.214) | (0.322) |
| Advice Quality (Low) |  |  | -3.542*** |
|  |  |  | (0.244) |
| Difficulty (Hard) |  |  | -0.136 |
|  |  |  | (0.206) |
| Difficulty (Very Hard) |  |  | -0.457* |
|  |  |  | (0.201) |
| Type (Probability) |  |  | -0.878*** |
|  |  |  | (0.187) |
| Type (Budget) |  |  | 0.173 |
|  |  |  | (0.194) |
| Random Effect SD | 1.429 | 0.718 | 1.074 |
| N | 2772 | 2772 | 2772 |
| logLik | -1407.563 | -874.492 | -652.054 |
| AIC | 2821.125 | 1760.983 | 1326.108 |
| *** p < 0.001; ** p < 0.01; * p < 0.05. |  |  |  |

**Table S8** Robust ANCOVA analysis of Algorithmic Advice on Confidence

| **Advice Quality: High** | | | | | | |
| --- | --- | --- | --- | --- | --- | --- |
| **Initial Confidence** | **Num Algorithm** | **Num Crowd** | **Difference** | **SE** | **Test Statistic** | **p-value** |
| Initial = 0 | 180 | 116 | 0.284 | 0.075 | 3.780 | 0.000 |
| Initial = 0.2 | 235 | 160 | 0.274 | 0.061 | 4.485 | 0.000 |
| Initial = 0.4 | 198 | 144 | 0.219 | 0.051 | 4.264 | 0.000 |
| Initial = 0.6 | 287 | 215 | 0.052 | 0.021 | 2.478 | 0.014 |
| Initial = 0.8 | 521 | 430 | 0.012 | 0.008 | 1.492 | 0.136 |
| Initial = 1 | 436 | 367 | 0.009 | 0.006 | 1.571 | 0.117 |
|  |  |  |  |  |  |  |
| **Advice Quality: Low** | | | | | | |
| **Initial Confidence** | **Num Algorithm** | **Num Crowd** | **Difference** | **SE** | **Test Statistic** | **p-value** |
| Initial = 0 | 208 | 116 | 0.140 | 0.032 | 4.331 | 0.000 |
| Initial = 0.2 | 289 | 156 | 0.166 | 0.034 | 4.863 | 0.000 |
| Initial = 0.4 | 287 | 106 | 0.128 | 0.043 | 2.961 | 0.004 |
| Initial = 0.6 | 374 | 158 | 0.055 | 0.019 | 2.967 | 0.003 |
| Initial = 0.8 | 518 | 430 | -0.003 | 0.011 | 0.241 | 0.810 |
| Initial = 1 | 444 | 360 | -0.008 | 0.009 | 0.863 | 0.388 |

*Notes. Difference is the trimmed mean difference between the two groups. SE is the corresponding standard error of the trimmed mean difference. Number of observations in each group is based on smoothing span of 1 (default in ANCOVA function).*

**SI References**

1. Bowden, E., & Jung-Beeman, M. (2003). Normative data for 144 compound remote associate problems. *Behavioral Research Methods, Instrumentation, and Computers,* 35, 634-639.
2. *Rhymezone*. Available at: https://www.rhymezone.com/ [Accessed July 8, 2021].
3. Nomura, T., Suzuki, T., Kanda, T., & Kato, K. (2006, July). Altered attitudes of people toward robots: Investigation through the Negative Attitudes toward Robots Scale. In *Proc. AAAI-*06 workshop on human implications of human-robot interaction (Vol. 2006, pp. 29-35).
4. Mair, P., & Wilcox, R. (2020). Robust statistical methods in R using the WRS2 package. *Behavior research methods*, *52*(2), 464-488.
5. Wilcox, R. (2012). Introduction to Robust Estimation and Hypothesis Testing (3rd ed.). Elsevier.
